# Gram-scale fermentative production of ergothioneine driven by overproduction of cysteine in *Escherichia coli*

**DOI:** 10.1038/s41598-018-38382-w

**Published:** 2019-02-13

**Authors:** Naoyuki Tanaka, Yusuke Kawano, Yasuharu Satoh, Tohru Dairi, Iwao Ohtsu

**Affiliations:** 1 Gradutate of School of Life and Environmental Sciences, University of Tsukuba, 1-1-1 Tennodai, Tsukuba, Ibaraki 305–8577 Japan; 2 Graduate School of Engineering, Hokkaido University, N13 & W8, Kita-ku, Sapporo, Hokkaido 060–8628 Japan

## Abstract

Ergothioneine (ERG), a unique thiol compound, is suggested to function as an antioxidant and cytoprotectant. Despite several recent attempts to produce ERG using various organisms, its yield was still very low and the costs remained high. Since the level of ERG produced depends strictly on the availability of three distinct precursor amino acids (l-cysteine (Cys), l-histidine, and l-methionine (Met)), metabolic engineering for enhancement of the flux toward ERG biosynthesis is required. Herein, we took advantage of a high-Cys production system using *Escherichia coli* cells, in which Cys biosynthesis and excretion were activated, and applied it to the fermentative production of ERG from glucose. The Cys overproduction in *E*. *coli* cells carrying the *egtBCDE* genes from *Mycobacterium smegmatis* was effective for ERG production. Furthermore, coexpression of the *egtA* gene, which encodes γ-glutamylcysteine synthetase that synthesizes the γ-glutamylcysteine used as a sulfur source of ERG biosynthesis, enhanced ERG production even though *E*. *coli* intrinsically has γ-glutamylcysteine synthetase. Additionally, disruption of the *metJ* gene that encodes the transcriptional repressor involved in Met metabolism was effective in further increasing the production of ERG. Finally, we succeeded in the high-level production of 1.31 g/L ERG in a fed-batch culture process using a jar fermenter.

## Introduction

To all living organisms, l-cysteine (Cys) is an essential amino acid that contributes to a number of biological processes, including oxidative stress tolerance and protein folding, assembly, and stability through disulfide-bond formation^1^. Cys is also used as a sulfur donor for the biosynthesis of important biological sulfur-containing molecules, such as glutathione, thiamine, and biotin^2,3^. Most microbes and plants can synthesize Cys from environmental inorganic sulfur sources. On the other hand, animals cannot assimilate inorganic sulfur sources, and instead obtain sulfur in the organic form as Cys and l-methionine (Met) through food intake. This implies that animals are completely dependent on bacterial and plant sulfur metabolites for their sulfur intake. In terms of this, organic sulfur-containing amino acids, including Cys, are industrially important. Bacterial fermentation is widely adopted for the mass production of many kinds of amino acids because of the benefits of industrial safety and cost effectiveness. In around 2000, the industrial-scale fermentative production of Cys was established by Wacker Chemie^4^. This fermentative approach, using *Escherichia coli*, is accomplished by the metabolic modification of some Cys biosynthetic enzymes (Supplementary Fig. S1); however, the sulfur utilization by this bacterium is poorly understood. Recently, we have demonstrated that *E*. *coli* prefers thiosulfate over sulfate for Cys biosynthesis^5,6^. This is because thiosulfate is advantageous for saving the consumption of NADPH and ATP molecules to synthesize Cys. That is, the sulfate pathway consumes two molecules of ATP and four molecules of NADPH as a reducing power to produce Cys from sulfate, whereas the thiosulfate pathway spends only one molecule of NADPH from thiosulfate. These facts led us to consider the potential advantage of converting overproduced Cys into beneficial Cys-derived compounds.

Ergothioneine (ERG), which is a betaine of 2-thiol-l-histidine, was first discovered in the ergot fungus *Claviceps purpurea*, and is suggested to be a unique sulfur-containing antioxidant, similar to glutathione, mycothiol, and bacillithiol^7^. Currently, ERG is known to be synthesized by limited kinds of microorganisms, such as mycobacteria (the order *Actinomycetales*), non-yeast-like fungi, and cyanobacteria^8–10^. Early studies carried out with cell-free extracts of *Neurospora crassa* demonstrated the requirement of three amino acids (viz., Cys, l-histidine (His), and l-methionine (Met)) for ERG biosynthesis^11,12^. Recently, Seebeck cloned five genes (*egtA*, *egtB*, *egtC*, *egtD*, and *egtE*) from *Mycobacterium smegmatis* and characterized their protein products *in vitro*, γ-GC) synthetase, catalyzes the formation of γ-GC from Cys and l-glutamate. EgtD, an *S*-adenosylmethionine (SAM)-dependent methyltransferase, mobilizes the methyl group from the substrate SAM and donates it to the amino group of His for the formation of hercynine (HER)^12–14^. HER and γ-GC are then converted into γ-glutamyl-hercynylcysteine sulfoxide via the oxidase hercynine oxygenase (EgtB), which requires oxygen and iron for its enzymatic activity^15,16^. Subsequently, EgtC, a glutamine amidotransamidase, facilitates the hydrolysis of the N-terminus glutamic acid, producing hercynylcysteine sulfoxide^17^. Finally, EgtE, a pyridoxal 5-phosphate-dependent beta-lyase, produces ERG, releasing pyruvate and NH_3_^13^.

Despite the limited range of organisms able to produce ERG, this amino acid derivative is also found in humans and other animals. Humans obtain ERG from their diet and accumulate it in various cells and tissues (e.g., kidney, liver, central nervous system, and red blood cells) at high concentrations (100 µM to 2 mM) through a highly specific ERG transporter, organic cation/carnitine transporter 1 (OCTN1)^7^. Several reports have proposed that ERG behaves as an antioxidant, scavenging hydroxyl radicals to prevent DNA damage and lipid peroxide formation^18–20^. The biodegradation of many sulfur compounds, including Cys, often results in the generation of hydrogen sulfide, which has an unpleasant odor^21,22^. On the other hand, ERG is not easily biodegraded, resulting in less unpleasant odors (our unpublished data). These unique properties of ERG are valuable for its use in a wide range of applications in the pharmaceutical, cosmetic, and food industries. Intact mushrooms (e.g., *Pleurotus ostreatus*) and cyanobacteria (e.g., *Spirulina*) have attracted attention as safe and accessible ERG sources^7,23^. However, their ERG contents are very low, at 0.4–2.0 mg/g dry weight in mushrooms and 0.8 mg/g dry weight in cyanobacteria^24,25^. In this respect, we recently showed that the high and heterologous expression of ERG enzymes of *M*. *smegmatis* in *E*. *coli* cells succeeded in producing 24 mg/L (104 µM) ERG in the broth supernatant^26^. The overproduction system in *Schizosaccharomyces pombe*, whose ERG biosynthetic pathway is different from that of *M*. *smegmatis*, resulted in an intracellular ERG yield of 370 mg/L (1.6 mM)^27^. However, further genetic and metabolic engineering studies are required to enhance the production of ERG.

In this study, using ERG biosynthetic genes from *M*. *smegmatis*, we applied a high-Cys-producing system to high ERG production and indeed showed its efficacy. We also propose that the biosynthetic activation of Met is key to the high-level production of ERG. Several further experiments to improve the biosynthetic flux of ERG succeeded in achieving its gram-scale production.

## Results and Discussion

**Figure 1:**
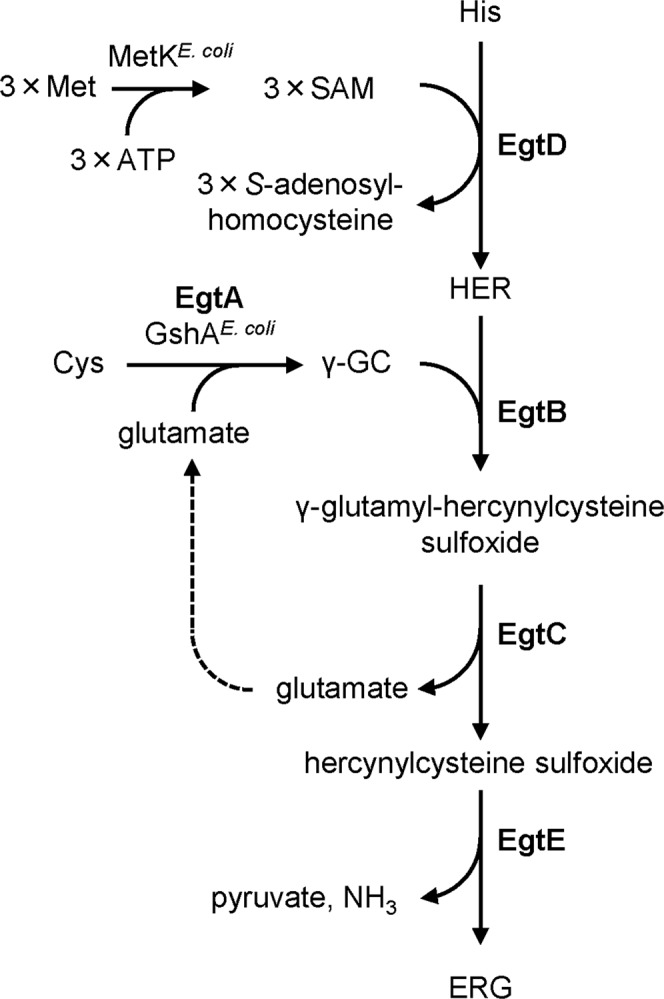
Biosynthetic pathway of ERG in *M*. *smegmatis*. ERG biosynthesis is mediated by five enzymatic steps. Expression of the foreign genes for EgtB, EgtC, EgtD and EgtE allows *E*. *coli* cells to synthesize ERG. EgtA from *M*. *smegmatis* is not indispensable because *E*. *coli* possesses GshA that catalyzes formation of γ-GC from Cys and glutamate. Met is used following formation of SAM by SAM synthetase (MetK in *E*. *coli*).

**Figure 2:**
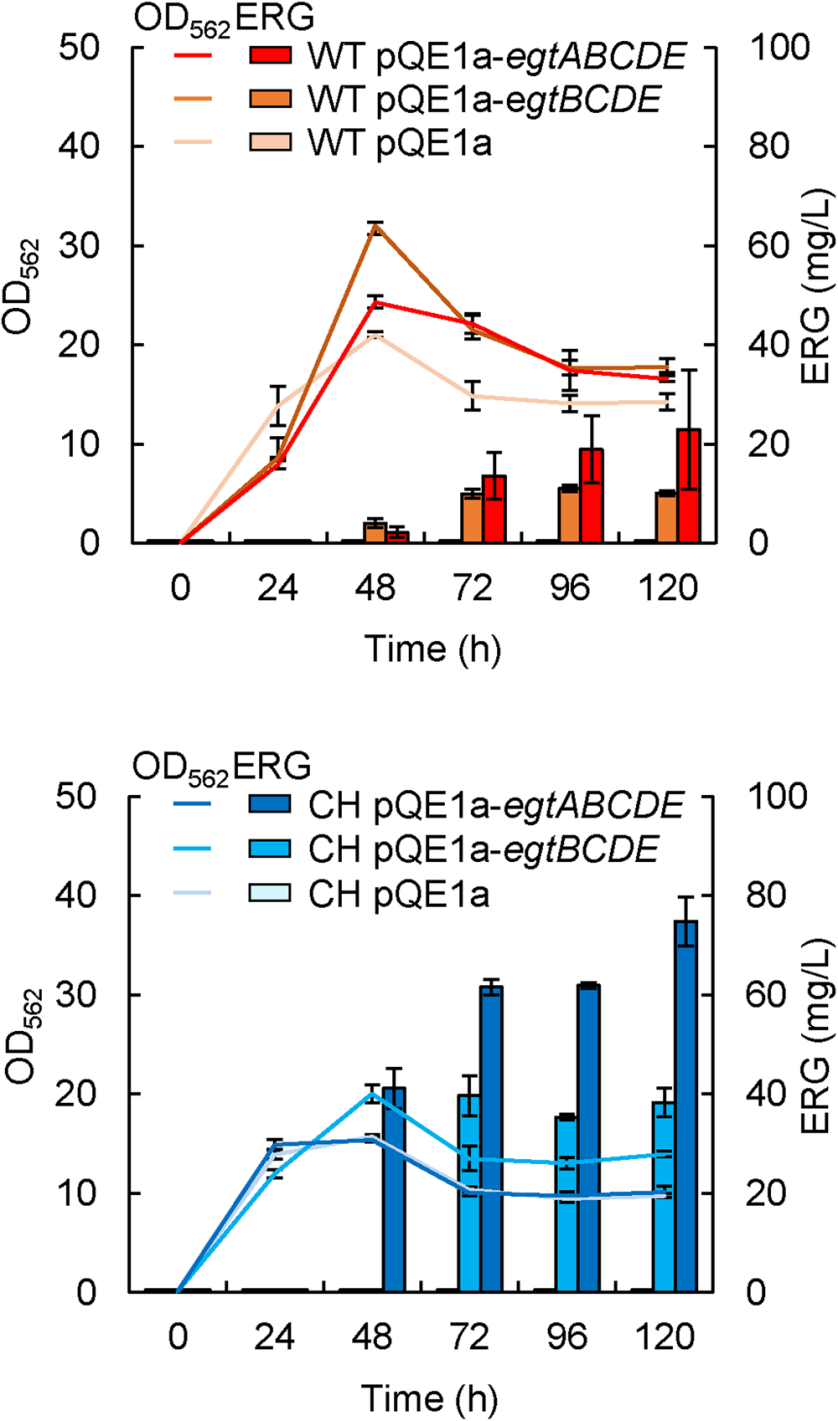
Expression of pCys^HP^ and *egtA* is remarkable efficient in ERG productivity. WT and CH harboring each of plasmids (pQE1a, pQE1a-*egtBCDE*, pQE1a-*egtABCDE*) were cultured in SM1 liquid medium. After 6 h cultivation, IPTG and Na_2_S_2_O_3_ were added at concentrations of 0.1 mM and 10 mM respectively. Data are presented as mean values with standard errors from three independent experiments. pACYC184 as negative control for pCys^HP^ was introduced into WT.

ERG production by a synthetic biological approach in *E*. *coli* and its enhancement by reinforcement of the sulfur metabolic flux toward γ-GC. In this study, we planned to establish a genetically engineered *E*. *coli* strain capable of high-level ERG production by a synthetic biological approach, since *E*. *coli* does not have ERG biosynthetic genes. Previously, we constructed three compatible plasmids into which each of *egt* genes (*egtB*, *egtC*, *egtD*, *egtE*) derived from *M*. *smegmatis* was cloned, and introduced them into *E*. *coli* strain to overproduce ERG enzymes in the cells^26^. These heterologous expressions allowed *E*. *coli* to biosynthesize ERG from inherently existing precursor metabolites, His, SAM, and γ-GC (Fig. 1), resulting in 24 mg/L ERG. In order to further improve the producing system for ERG, we here constructed the plasmid pQE1a-*egtBCDE*, capable of facilitating the isopropyl β-D-1-thiogalactopyranoside (IPTG)-inducible polycistronic expression of the *egtBCDE* derived from *M*. *smegmatis* in *E*. *coli*. The plasmid was introduced into the wild-type (WT) *E*. *coli* K-12 BW25113 strain to give WT pQE1a-*egtBCDE*. For the sake of facilitating later comparison with another genetic engineering construction (CH pQE1a-*egtBCDE*, mentioned below), a negative-control plasmid (pACYC184)^28^ was also introduced. We then examined whether this strain could produce extracellular ERG (Fig. 2). As a result, WT pQE1a pACYC184 did not produce ERG at all, whereas WT pQE1a-*egtBCDE* pACYC184 successfully produced 10 mg/L of ERG after 120 h of cultivation, indicating the successful genetic design and construction for ERG production based on the heterologous expression of *egtBCDE* from *M*. *smegmatis*.

To further increase the ERG yield, we focused next on reinforcing the sulfur metabolic flux toward ERG biosynthesis, because this pathway was indicated to be the rate-limiting pathway in our previous study^26^. First, we attempted genetic engineering for a more effective biosynthesis of Cys. Previously, we introduced the plasmid pDES into the *E*. *coli* cells and found a remarkable production of Cys^5,6,29^. The plasmid pDES contains three genes from *E*. *coli*, the altered *cysE* gene encoding the Cys feedback inhibition-insensitive mutant SAT (T167A)^30^, the wild-type *ydeD* gene encoding inner membrane Cys exporter, and the altered *serA* gene encoding the l-serine feedback inhibition-insensitive mutant of d-3-phosphoglycerate dehydrogenase (T410stop). Expression of each gene is controlled by the constitutive promoter of the *E*. *coli ompA* gene encoding outer membrane protein A precursor. To adapt this high-Cys producing system for the purpose of ERG production, we redesigned and constructed plasmid pCys^HP^ based on the pDES. WT pCys^HP^ could produce a large amount of Cys (Supplementary Fig. S2), and we designated this strain as “CH” (Cys hyperproducer). Remarkably, CH pQE1a-*egtBCDE* produced 40 mg/L of ERG after 120 h of cultivation, which was a 4-fold higher yield than that from WT pQE1a-*egtBCDE* pACYC184 (Fig. 2). This showed that the reinforcement of Cys biosynthesis is extremely effective for ERG production. In order to test the effect of lack of *ydeD* gene on ERG production, we constructed the plasmid carrying the altered *cysE* gene and the altered *serA* gene by excision of the *ydeD* gene from the pCys^HP^, and then introduced it into the WT cells. No significant effect was observed on ERG production even though the amount of Cys decreased (data not shown). This result suggests that Cys production level without YdeD was adequate for production of ERG under our conditions at present. After this, we employed the pCys^HP^ in all experiments because of the higher Cys production.

We subsequently attempted to enhance the Cys-to-γ-GC step. To this end, we constructed another plasmid, pQE1a-*egtABCDE*, capable of coexpressing the *egtA* gene of *M*. *smegmatis*. In this ERG biosynthesis, the γ-GC biosynthetic step is catalyzed by exogenous EgtA and endogenous glutamate-cysteine ligase (GshA) in *E*. *coli*. Notably, CH pQE1a-*egtABCDE* could produce 75 mg/L of ERG after 120 h of cultivation (Fig. 2), which was a 2-fold higher yield than that from CH pQE1a-*egtBCDE*. This result indicated that EgtA coexpression contributed to enhancement of the γ-GC supply and subsequently higher ERG production.

γ-GC can be utilized in both biosynthetic pathways for glutathione and ERG. In *E*. *coli*, glutathione synthetase (GshB) is responsible for the catalytic synthesis of glutathione from γ-GC and glycine^31,32^. To facilitate the γ-GC supply toward ERG biosynthesis, we examined the effect of deletion of the GshB-encoding gene on ERG yield. Unexpectedly, no effect was seen on ERG production in the Δ*gshB* strain compared with the WT under our experimental conditions (Supplementary Fig. S3). It should be noted that the ERG produced could not complement the slow growth phenotype of Δ*gshB* caused by the lack of glutathione^33^. Glutathione is known to play major roles in the intracellular antioxidative system in eukaryotes and some bacteria, including *E*. *coli*^3^. Because thiol compounds generally function as a reducing agent, ERG also plays a role such as Cys and glutathione. Although, there is no evidence for certain *in vivo* function of ERG. Several critical *in vitro* studies have demonstrated unique properties of ERG that are different from those of any other thiol compounds: (i) at normal physiological pH, ERG exists predominantly in the thione form rather than the thiol form^7^; (ii) ERG demonstrates a greater deactivation effect on singlet oxygen and hydroxyl radicals than other thiols, including glutathione^18^; and (iii) ERG has poor activity against hydrogen peroxide and superoxide^18,19^. These properties and our results indicate the different roles of ERG and glutathione (and other thiols) *in vivo*. Hydrogen peroxide and superoxide are scavenged by antioxidative enzymes, such as catalase and dismutase, as well as antioxidative thiols. However, cells do not have endogenous enzymes and compounds that can eliminate hydroxyl radicals and singlet oxygen directly. ERG might therefore play a role as the direct and sole scavenger of hydroxyl radicals.

### Effect of metJ gene disruption on ERG production

**Figure 3:**
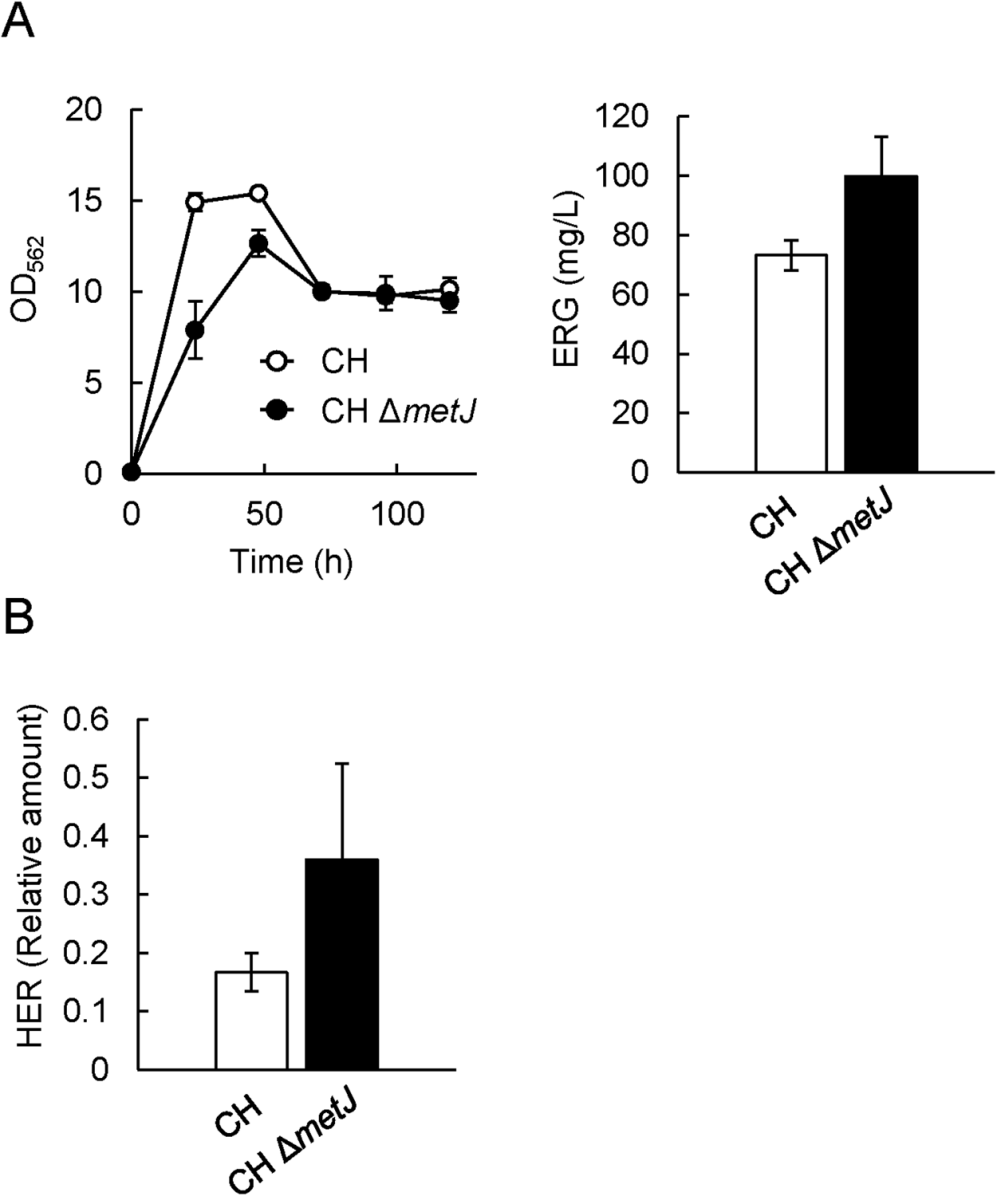
Impact of *metJ* gene disruption on ERG and HER productivities. (**A**) CH pQE1a-*egtABCDE* and CH Δ*metJ* pQE1a-*egtABCDE* were cultured in SM1 liquid medium. After 6 h cultivation, IPTG and Na_2_S_2_O_3_ were added at concentrations of 0.1 mM and 10 mM respectively. Cell density (left) was estimated from the OD at 562 nm. ERG (right) in the cultured supernatant at 120 h was quantified by Sulfur index analysis described in Methods. (**B**) WT and Δ*metJ* cells harboring pCys^HP^ and pCF1s-*egtD* were cultured in SM1 liquid medium. After 6 h cultivation, IPTG and Na_2_S_2_O_3_ were added at concentrations of 0.1 mM and 10 mM respectively. HER in the cultured supernatant at 120 h was quantified by LC-MS/MS analysis. Data are presented as mean values with standard errors from three independent experiments.

HER is synthesized from His and SAM by EgtD, which is a SAM-dependent methyltransferase (Fig. 1). SAM synthetase is the enzyme that catalyzes the conversion of Met and ATP into SAM. Since the synthesis of one HER molecule requires one His molecule and three Met molecules, it is very likely that the biosynthetic steps of Met and SAM are rate-limiting in ERG production. The transcriptional levels of various genes encoding enzymes in the Met and SAM biosynthetic pathways are controlled by the transcriptional repressor MetJ^34^. This repressor also represses the gene encoding MetR, which is a transcriptional activator responsible for several Met biosynthetic genes. We therefore focused on MetJ and assessed the impact of *metJ* gene disruption on the ERG productivity of the mutant strain. The pCys^HP^ and pQE1a-*egtABCDE* plasmids were introduced into Δ*metJ* cells (termed CH Δ*metJ* pQE1a-*egtABCDE*), and the ERG productivity of the strain was evaluated. The Δ*metJ* cells occurred in growth defect in the fermentative conditions (Fig. 3A). By contrast, CH Δ*metJ* pQE1a-*egtABCDE* produced a higher amount of ERG (98 mg/L at 120 h) than CH pQE1a-*egtABCDE* did. These results indicated that *metJ* disruption is effective in improving the ERG yield, probably due to improvement of the biosynthetic steps toward Met and subsequently SAM and HER. In fact, when pCF1s-*egtD*, which allows the cells to synthesize HER^26^, was introduced into WT and Δ*metJ* cells, HER production was higher in the Δ*metJ* cells than in the WT (Fig. 3B). According to the EcoCyc database (https://ecocyc.org/), MetJ regulates the transcriptional expression of 15 genes (Table 1). Of these, *metF* (encoding methylenetetrahydrofolate reductase, involved in Met biosynthesis) and *metK* (the SAM synthetase gene)^35^ are known to exhibit higher transcriptional levels in Δ*metJ* than in the WT^36^. Thus, in our case of ERG production, such higher transcription levels and resulting activation of MetF and MetK in Δ*metJ* are considered to be a trigger in facilitating HER production and consequently ERG production.

**Table 1:** Genes regulated by transcriptional repressor MetJ in *E. coli*.

Gene	Function
*metA*	Homoserine *O*-succinyltransferase,
*metB*	*O*-succinylhomoserine(thiol)-lyase/*O*-succinylhomoserine lyase
*metC*	cystathionine-β-lyase/Cys desulfhydrase
*metE*	cobalamin-independent homocysteine transmethylase
*metF*	5,10-methylenetetrahydrofolate reductase
*metI*	L-methionine/D-methionine ABC transporter membrane subunit
*metK*	methionine adenosyltransferase
*metL*	fused aspartate kinase/homoserine dehydrogenase 2
*metN*	L-methionine/D-methionine ABC transporter ATP binding subunit
*metR*	DNA-binding transcriptional regulator involved in methionine biosynthesis
*metQ*	L-methionine/D-methionine ABC transporter membrane anchored binding protein
*ahpC*	alkyl hydroperoxide reductase, AhpC component
*ahpF*	alkyl hydroperoxide reductase, AhpF component
*folE*	GTP cyclohydrolase 1
*yeiB*	DUF418 domain-containing protein YeiB

### Optimization of the fermentative conditions for ERG production

**Figure 4:**
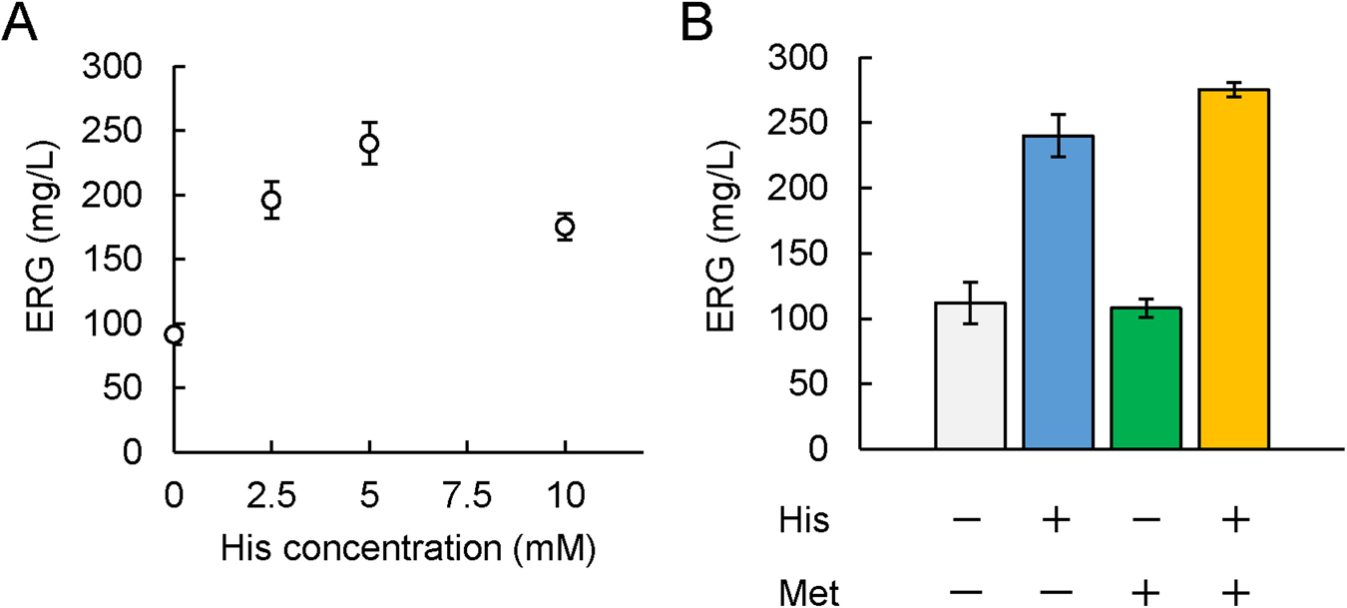
Effect of supplementation with His and Met on ERG productivity. (**A**) CH Δ*metJ* pQE1a-*egtABCDE* cells were cultured in SM1 liquid medium. After 6 h cultivation, His was added at various concentrations (0, 2.5, 5, 10 mM) together with IPTG, Na_2_S_2_O_3_ at concentrations of 0.1 mM and 10 mM respectively. ERG in the cultured supernatant at 120 h was quantified by Sulfur index analysis described in Methods. (**B**) CH Δ*metJ* pQE1a-*egtABCDE* cells were cultured in SM1 liquid medium. After 6 h cultivation, His, Met, or their combination was added at concentrations of 5 mM and 3 mM, respectively, together with IPTG, Na_2_S_2_O_3_ at concentrations of 0.1 mM and 10 mM respectively. ERG in the cultured supernatant at 120 h was quantified by Sulfur index analysis described in Methods.

As His is the precursor of HER, we examined the effect of supplementation of His into the fermentation medium on ERG production. As shown in Fig. 4A, the supplementation of His enhanced ERG production significantly, resulting in 240 mg/L ERG at the His concentration of 5 mM. We therefore supplemented His at concentration 5 mM into the growth medium in following flask-scale experiments. In order to increase the amount of HER, we also examined the effect of an increase of Met concentration in the medium. The addition of Met only had no effect on ERG production (Fig. 4B). We speculated insufficient supply of His to accept methyl groups derived from Met for the enhancement of HER production. Predictably, the combined addition of Met and His had an additive effect on the ERG productivity (275 mg/L) of the cells (Fig. 4B). These results suggest the significance and effectiveness of exceeding the supply of the precursors to overproduce ERG, but at the same time, implying the requirement of the further improvement of metabolic engineering to supply His and Met.

To facilitate the ERG productivity of *E*. *coli* further, we examined the effects of adding several supplements (e.g., minerals, amino acids, vitamins) into the fermentation medium and found that the supplementation of iron, in particular ammonium ferric citrate (AFC), had a significant effect on the stable production of ERG. In the presence of up to 5 mg/L AFC, ERG production increased in a concentration-dependent manner (Supplementary Fig. S4). This iron effect on ERG production is consistent with a previous report using *Methylobacterium* species^27^. In ERG biosynthesis, EgtB is dependent on iron for its catalysis^16^. Thus, a high ferric concentration might activate the enzymatic reaction mediated by EgtB. These observations suggest the potential for further refinement of the biosynthetic step by EgtB for ERG overproduction.

### ERG production in a fed-batch culture

**Figure 5:**
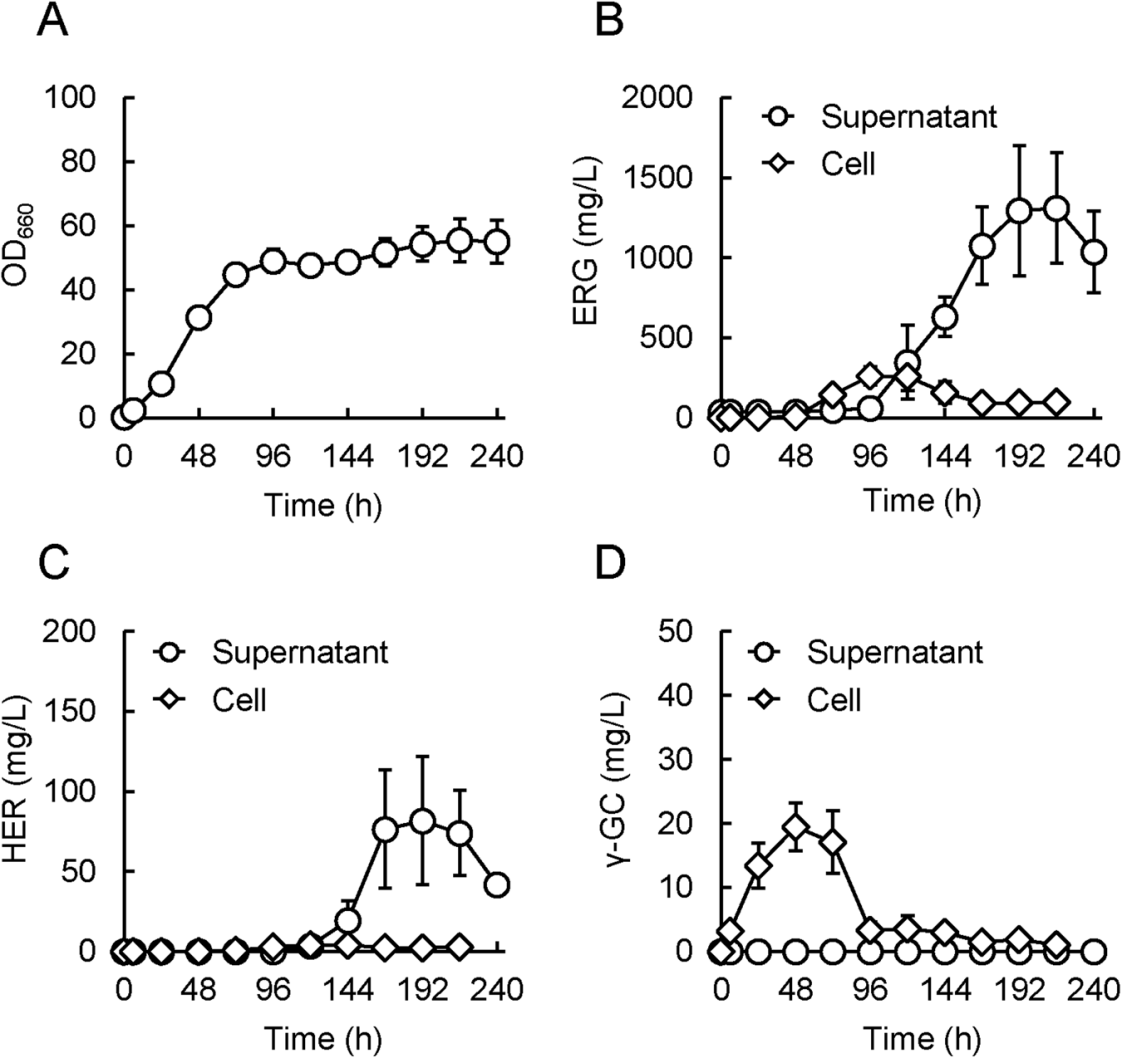
ERG production in a fed-batch culture. Growth (**A**), ERG production (**B**), HER production (**C**), and γ-GC production (**D**) of CH Δ*metJ* pQE1a-*egtABCDE* cells in the fed-batch culture by a jar fermenter. Growth was monitored by measuring OD_660_ of the culture cell suspension. ERG, HER, and γ-GC were measured by Sulfur index and the LC-MS/MS analysis described in Methods. For the measurements of “cell”, its metabolites were extracted beforehand, and utilized for measurements (described in Methods). For concentrations of indicated compounds in cell were calculated considering cell density in the culture at each time point. Data are presented as mean values with standard errors from three independent experiments.

To attempt further enhancement of the ERG yield, we applied a jar fermenter to accomplish a higher cell density and thus effective ERG production, using the fed-batch technique with supply of glucose and precursors of ERG biosynthesis, respectively. The composition of the culture medium was based on the conditions investigated in the flask experiments above, but some components were prepared at somewhat higher concentrations (see Methods), taking into consideration the aim for a higher cell density in this culture system. Since EgtE contains a pyridoxal 5-phosphate as a cofactor^13^, enough pyridoxine (a precursor of pyridoxal 5-phosphate) was added to the growth medium in this experiment. In brief, the design of the culture process was based on the following ideas: at 0–24 h, no substrate was fed, with emphasis on a favorable onset of cell growth; at 24–48 h, only glucose was fed, with emphasis on achieving the highest cell density possible beforehand; at 48 h, IPTG, additional AFC, and pyridoxine were added to initiate ERG production; and at 48–120 h, glucose and precursors of ERG biosynthesis (Met, His, and thiosulfate) were constantly fed, with emphasis on persistent and effective ERG production. In this experiment, CH Δ*metJ* pQE1a-*egtABCDE*, the best ERG producer in the flask experiments mentioned above, was employed. Consequently, the cells grew well, with the OD_660_ reaching ~40 in 72 h and finally ~55 (Fig. 5A). ERG production in the supernatant increased from 120 h and reached 1,311 mg/L at 216 h (Fig. 5B). ERG production in the cells increased from 72 h, reached 261 mg/L at 96 h, and decreased gradually thereafter (Fig. 5B). HER also accumulated in the supernatant in an almost similar time course to that of ERG production, but importantly, its concentration was much less than that of ERG (~1/20) at 216 h (Fig. 5C). Additionally, γ-GC accumulated in the cells mainly in the earlier period (24–72 h), but its level decreased in the period of ERG production (96 h onward) and the resulting level was likewise much less than that of ERG at 216 h (Fig. 5D). This decrease of γ-GC indicates that pre-accumulated γ-GC was appropriately consumed for the induction of ERG production. Additionally, γ-glutamyl-hercynylcysteine sulfoxide and hercynylcysteine sulfoxide, which are intermediates between HER and ERG (Fig. 1), were hardly detectable during the culture, both in the cell and supernatant (data not shown). These results suggest that there were no critical rate-limiting steps in the ERG biosynthetic metabolic flow in this strain. Taken together, both our genetic construction and culture design were considered to have worked well. Notably, most of the ERG produced actually existed extracellularly (>93%) at 216 h. This trait should be preferable for purification of the ERG produced, which is necessary for practical ERG manufacturing in an industrial process, which is the goal of this study.

Interestingly, ERG production in the supernatant was slightly delayed compared with that in the cells, corresponding to the induction of pQE1a-*egtABCDE* by IPTG addition at 48 h. It is possible that such delay and subsequent acceleration of extracellular ERG production might have involved an unknown ERG-exporting transporter. It should be noted that such ERG transporter is to our best knowledge currently unidentified in all biological species, although *Mycobacterium* species is reported to actively export ERG extracellularly^37^. Because *E*. *coli* itself does not have known ERG biosynthetic genes, and in fact does not produce ERG, such export might occur via a transporter for other structurally similar molecules, perhaps in a low specific manner. The exploration and identification of such ERG exporter gene and its application to appropriate genetic engineering would be a significant and promising approach to further accelerate and/or enhance the ERG productivity of bacterial cells.

## Conclusions

Unlike Cys and glutathione, ERG does not release a sulfur odor, and is a biologically important thiol against hydroxyl radicals^7,18,19^. Its dietary intake is likely to reduce hydroxyl and other radicals safely and circumvent DNA damage and fatty acid peroxidation products, thereby inhibiting carcinogenesis, lifestyle-related chronic diseases, and aging^7^. Although an inexpensive and safe fermentation technique for ERG production using microbes is desired, Cys biosynthesis and its metabolic flux toward ERG biosynthesis (the rate-limiting steps in ERG biosynthesis) have hampered the hyperproduction of ERG. Recently, our technique for Cys fermentative production has reached the level of practical application (>16 g/L, our unpublished data), although further improvement is still needed for cost-effectiveness. This led us to the idea of applying Cys overproduction to an ERG-hyperproducing system. The present study has demonstrated that the synthetic biological approach of facilitating the heterologous expression of ERG biosynthetic genes from *M*. *smegmatis* in *E*. *coli* cells is practical and viable, allowing the synthesis of ERG from the precursors His, SAM, and γ-GC. In addition, reinforcement of the γ-GC supply, by both genetic modification of the Cys biosynthetic pathway and additional expression of EgtA, was shown to be effective in increasing the ERG productivity of the cells. Similarly, reinforcement of the SAM supply by disruption of the transcriptional repressor MetJ was most effective as well. Importantly, we have, for the first time, successfully produced ERG on the gram scale (1.31 g/L) using the fed-batch culture technique. We believe that the present study will be a milestone by providing a practical approach to overproduce ERG in the industrial setting.

## Methods

### Bacterial strains and cell growth

The *E*. *coli* strains used in this study are listed in Table 2^38^. LB medium was used as the standard medium. For fermentative production, the cells were grown in SM1 minimal medium (1 g/L Trypton, 0.5 g/L Yeast extract, 100 µM potassium phosphate buffer (pH 7.0), 75.7 µM (NH_4_)_2_SO_4_, 1.7 µM NaCl, 1.0 µM MgSO_4_, 0.1 µM CaCl_2_, 20 mg/L ammonium ferric citrate (AFC), 0.6 µM Na_2_MoO_4_, 40.4 µM H_3_BO_3_, 2.9 µM CoCl_2_, 1 µM CuSO_4_, 8.1 µM MnCl_2_, 3.04 mM Met) supplemented with 3% glucose. When required, ampicillin (Ap), tetracycline (Tc), kanamycin (Km) and streptomycin (Sm) were added at concentrations of 50, 10, 50 and 50 µg/ml, respectively. Cultures were incubated aerobically by shaking at 30 °C. Growth was monitored by measurement of the optical density at 562 nm or 660 nm using U-1100 Spectrophotometer (HITACHI).

**Table 2:** *E*. *coli* strains used in this study.

Strain	Genotype	Reference or source
BW25113	Wild type, *rrnB3* ∆*lacZ4787 hsdR514* ∆(*araBAD*)567 ∆(*rhaBAD*)568 *rph-1*	^38^
JW3909	BW25113 ∆*metJ*::Km^r^	^38^

### Plasmid construction

Plasmids used in this study are listed in Table 3. Gene cluster including *egtBCDE* genes (MSMEG_6246 to 6249) was amplified by PCR using genomic DNA of *M*. *smegmatis*, primers (5′-GGAGATATACATATGATCGCACGCGAGACACTGGCCGACGAG-3′ and 5′-TATAAGCTTCAGGGCGCCTCACGCAACGCTG-3′), and Tks Gflex DNA polymerase (Takara Bio. Inc., Shiga, Japan). The amplified DNA fragment was digested with *Nde*I and *Hind*III, and then inserted into the corresponding sites of pQE1a-Red to obtain pQE1a-*egtBCDE*. To express *egtABCDE* gene cluster (MSMEG_6246 to 6250), pQE1a-*egtBCDE* was modified. A DNA fragment including *egtA* was amplified by PCR using genomic DNA of *M*. *smegmatis*, primers (5′-GGAGATATACATATGGCCTTACCCGCCAGAAGTGATTCTG-3′ and 5′-CGTGCTGTAGCGCTCGTAGATCATC-3′), Tks Gflex DNA polymerase. The fragment obtained was digested *Nde*I and *Afe*I and inserted into the corresponding sites of pQE1a-*egtBCDE* to obtain pQE1a-*egtABCDE*.

**Table 3:** Plasmids used in this study.

Plasmid	Description	Reference or source
pACYC184	Cm^r^, Tc^r^, p15A replicon	^28^
pCys^HP^	Tc^r^, pACYC184 derivative carrying *serA* (T410 stop), *ydeD and cysE* (T167A, G203S, T234S, P252L, M256Q) from *E*. *coli* under the control of the *ompA* promoter	^29^, This study
pQE1a-Red	Ap^r^, ColE1 replicon, *tac* promoter	^26^
pQE1a-*egtBCDE*	pQE1a-Red derivative carrying *M*. *smegmatis egtBCDE*	This study
pQE1a-*egtABCDE*	pQE1a-Red derivative carrying *M*. *smegmatis egtABCDE*	This study
pCF1s-*egtD*	Sm^r^, pCF1s-Red derivative carrying *M*. *smegmatis egtD*	^26^

### Fermentative production in flask scale

*E*. *coli* cells were grown in SM1 medium supplemented with a saturated amount of CaCO_3_ at 30 °C with shaking. At 6 h, Na_2_S_2_O_3_ and IPTG were added at 10 mM and 0.1 mM, respectively. To evaluate the growth, the cell suspension containing insoluble CaCO_3_ was added to 0.1 N HCl to dissolve CaCO_3_, after which the OD_562_ was measured. To determine the ERG concentration in the medium, the cell suspension was centrifuged, and the supernatant was appropriately diluted with H_2_O. For the reduction of ERG, the diluted supernatant was incubated in in 50 mM Tris-HCl buffer (pH 8.5) containing 5 mM dithiothreitol for 10 min at room temperature. The concentration of ERG was determined by Sulfur index analysis described below. For experiments of HER fermentative production, we used the *E*. *coli* strain harboring the plasmid pCF1s-*egtD*^26^ carrying *egtD* gene because of easy and sensitive detection of HER. HER levels were determined by LC-MS/MS analysis. Cys concentration was conducted by Gaitonde’s method as described^39^.

### Sulfur index analysis

The supernatants of cell culture were subjected to analysis of Sulfur index, which is defined as a profile of major sulfur metabolite contents, and measured by the sulfur metabolomic method^6^. Sulfur index is a sulfur-metabolomics by using combination of LC-MS/MS and thiol-specific derivatization method using monobromobimane. In this method, derivatized sulfur metabolites (e.g., Cys, γ-GC, and ERG) were reliably detected and quantified by the LC-MS/MS system. The target metabolite levels were determined from the peak area by mass chromatography, monitoring each m/z characteristic to the individual target, and were represented as relative amounts after normalization with the peak area of the internal standard (d-camphor-10-sulfonic acid).

### ERG production in a fed-batch culture

*E. coli* cells were precultured overnight in LB medium at 30 °C using a baffled flask with rotary shaking. The cells were centrifugally collected, resuspended with 20 mL LB medium, and used as the inoculum. For the main culture performed in a 3-L jar fermenter (MBC-3; Sanki Seiki, Osaka, Japan), 1.1 L of the medium was prepared, which contained the following compounds; 23.24 g K_2_HPO_4_, 9.06 g KH_2_PO_4_, 4 g tryptone, 2 g yeast extract, 1.2 g NaCl, 20 g (NH_4_)_2_SO_4_, 0.9 g Met, 60 mg AFC, 0.248 mg Na_2_MoO_4_, 5 mg H_3_BO_3_, 0.754 mg CoCl_2_, 0.32 mg CuSO_4_, 2.04 mg MnCl_2_, 240 mg MgSO_4_, 22.2 mg CaCl_2_, 40 g glucose, and 3 mL Antifoam SI (Wako Pure Chemical Industries). After preparing pH of the medium (~6.8) to 6.9 by adding ammonia solution (28%) (Nacalai Tesque), the culture was started by adding the above-mentioned inoculum into the medium. The culture was performed at 30 °C, agitated at 490 rpm by two blade-type turbine impellers, and aerated at 1 L/min. The pH of the culture medium was monitored and kept between 6.9–7.0 by automatic addition of ammonia solution (28%) (Nacalai Tesque) or 25% (v/v) H_2_SO_4_. From 24 to 48 h, 400 g/L glucose was constantly fed to the culture medium (58.3 µL per 12.6 sec; totally 400 ml). At 48 h, 2 mL of 10 g/L pyridoxine-HCl, 1 mL of 30 g/L AFC, and 2 mL of 0.1 M IPTG were added. From 48 to 120 h, 400 g/L glucose was constantly fed (58.3 µL per 60.5 sec; totally 250 mL), and another mixed solution (18.2 g/L His-HCl monohydrate, 39.1 g/L Met, and 1.58 g/L Na_2_S_2_O_3_) was also fed constantly (58.3 µL per 60.5 sec; totally 250 mL). After completion of all the feedings mentioned, culture volume reached approximately 2 L. From 72 to 240 h, 1 ml of Antifoam SI was added every day. At the indicated time points, the cell suspension was sampled and its OD_660_ was measured to evaluate the growth after appropriate dilution with H_2_O. The supernatant samples were obtained after centrifugation of the suspension. The cell samples were obtained after centrifugation of the suspension and subsequent removal of its supernatant. The supernatant samples were appropriately diluted with H_2_O. The cell samples were resuspended with 80% (v/v) methanol and its metabolites including ERG were extracted from the cells by bath sonication treatments. After centrifugation, supernatant was obtained, dried up by centrifugal evaporator, resuspended with H_2_O water, centrifuged again, and its supernatant was utilized as the cell extract sample.

## Supplementary information

SUPPLEMENTARY INFO

## Data Availability

The datasets generated during the current study are available from the corresponding author on reasonable request.
