# Supplementary material for: Gram-scale fermentative production of ergothioneine driven by overproduction of cysteine in Escherichia coli
Source: Sci Rep. 2019 Feb 13;9:1895. doi: 10.1038/s41598-018-38382-w (PMC6374457; doi:10.1038/s41598-018-38382-w)
Supplement: Supplementary file 1 — SUPPLEMENTARY INFO [file 41598_2018_38382_MOESM1_ESM.pdf]

Supplementary Information

Gram-scale fermentative production of ergothioneine driven by overproduction of cysteine in *Escherichia coli*.

Naoyuki Tanaka[1], Yusuke Kawano[1], Yasuharu Satoh[2], Tohru Dairi[2] and Iwao Ohtsu[1]*

[1]*Graduate School of Life and Environmental Sciences, University of Tsukuba, 1-1-1 Tennodai, Tsukuba, Ibaraki, 305-8577, Japan*

[2]*Graduate School of Engineering, Hokkaido University, N13 & W8, Kita-ku, Sapporo, Hokkaido 060-8628, Japan.*

* Corresponding author

Figure S1. Sulfur assimilation pathways in *E. coli*. p2

Figure S2. Cys production in *E. coli*. p3

Figure S3. ERG production in Δ*gshB* background. p4

Figure S4. Effect of supplementation with iron on ERG productivity. p5

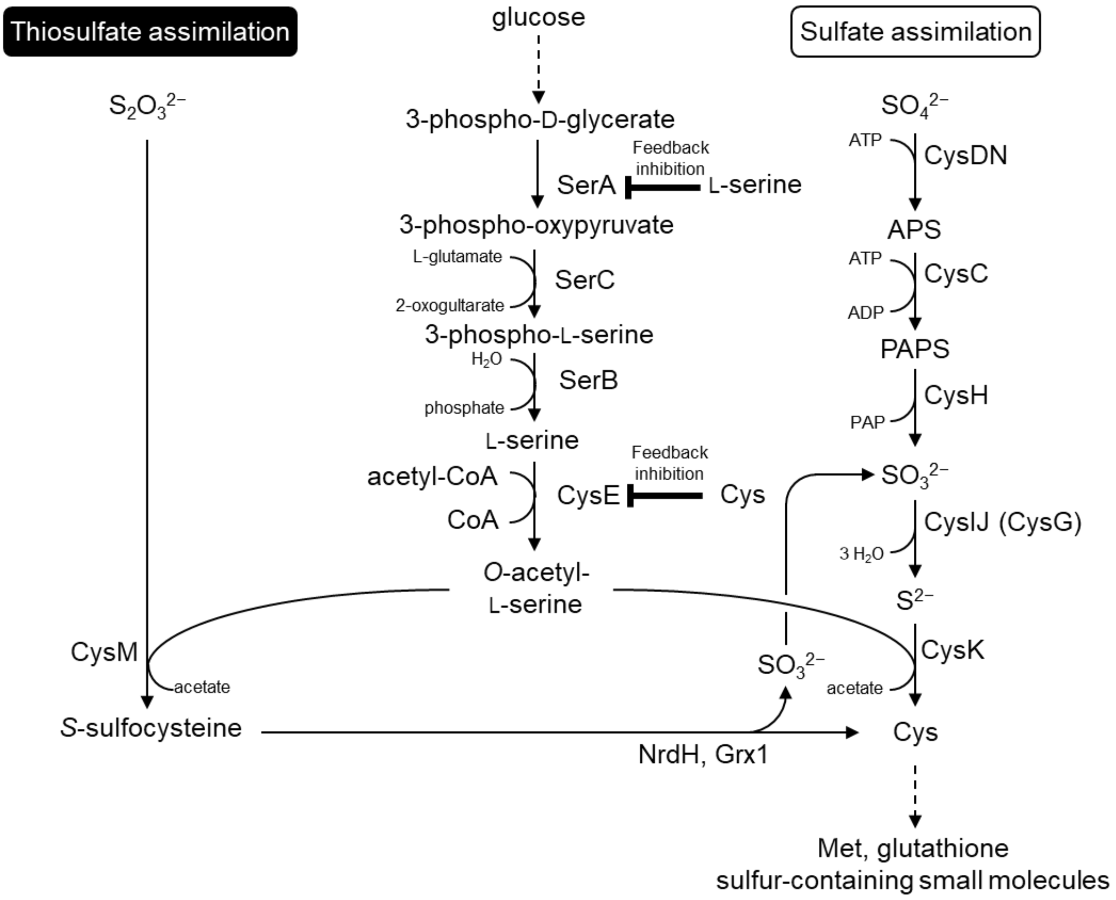

Figure S1. Sulfur assimilation pathways in *E. coli*.

E. coli possesses two pathways for sulfur assimilation: one is sulfate pathway, the other is thiosulfate pathway. In both pathways, the carbon metabolite accepting the sulfur atom is commonly *O*-acetyl-L-serine. In the sulfate pathway, *O*-acetyl-L-serine sulfhydrylase A (CysK) catalyzes the conversion of *O*-acetyl-L-serine and sulfide into Cys. In the thiosulfate pathway, *O*-acetyl-L-serine sulfhydrylase B (CysM) catalyzes the conversion of *O*-acetyl-L-serine and thiosulfate into *S*-sulfocysteine. *S*-sulfocysteine is subsequently metabolized into Cys by NrdH and Grx1, and the simultaneously released sulfite is also assimilated into another Cys via the sulfate pathway. SerA and CysE are controlled through feedback inhibition by serine and Cys, respectively. $S_2O_3^{2-}$, thiosulfate; SO_4^{2-}, sulfate; SO_3^{2-}, sulfite; S^{2-}, sulfide; APS, adenosine 5'-phosphosulfate; PAPS, 3'-phosphoadenosine 5'-phosphosulfate; Grx, glutaredoxin.

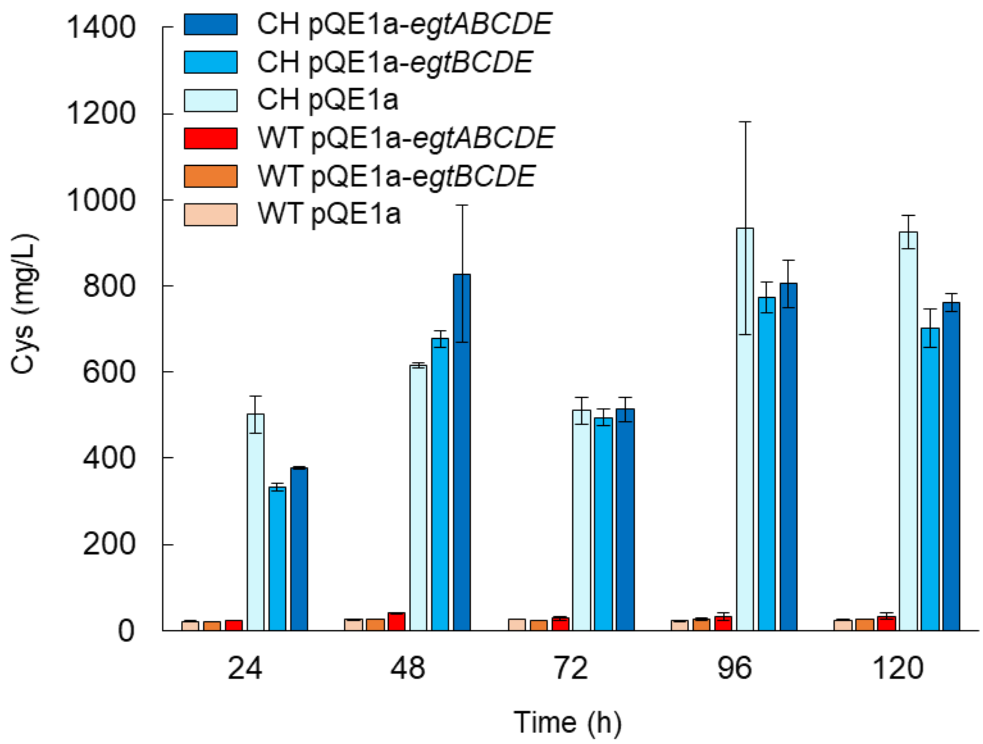

Figure S2. Cys production in *E. coli.*

WT and CH harboring each of plasmids (pQE1a, pQE1a-*egtBCDE*, pQE1a-*egtABCDE*) were cultured in SM1 liquid medium. After 6 h cultivation, IPTG and $Na_2S_2O_3$ were added at concentrations of 0.1 mM and 10 mM respectively. Cys in the cultured supernatant was determined by Gaitonde's method (described in Methods). Data are presented as mean values with standard errors from three independent experiments. pACYC184 as negative control for $pCys^{HP}$ was introduced into WT.

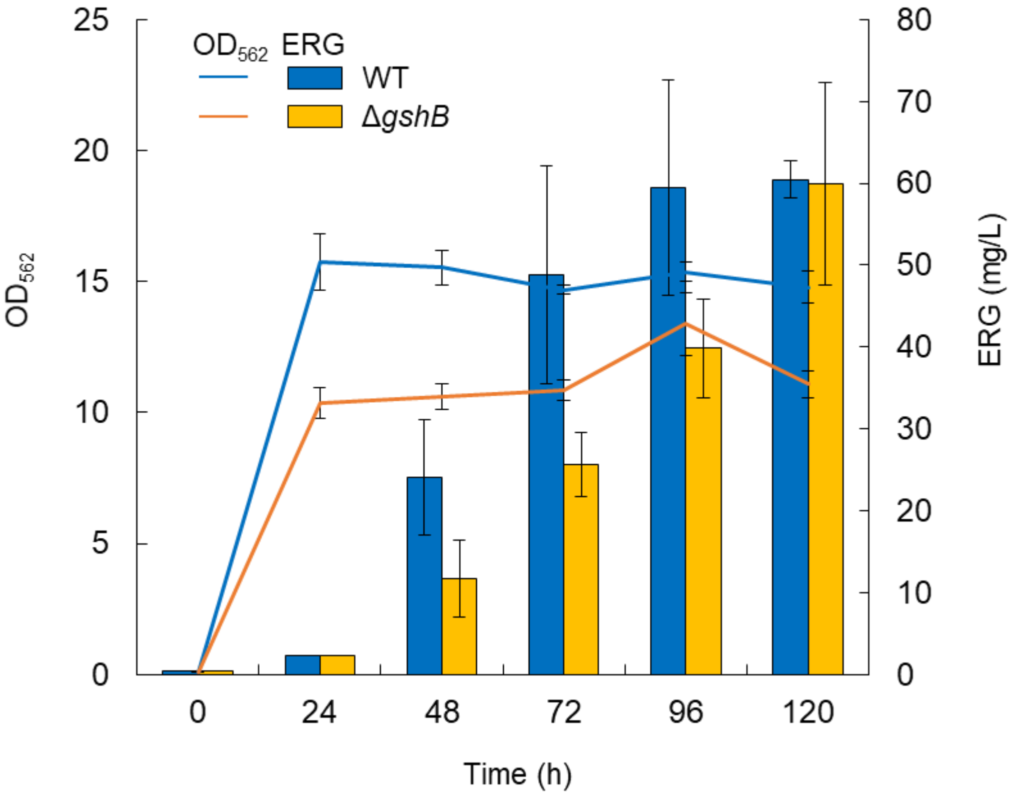

Figure S3. ERG production in Δ*gshB* background.

WT and Δ*gshB* cells harboring pCys[HP] and pQE1a-*egtABCDE* were cultured in SM1 liquid medium. After 6 h cultivation, IPTG and $Na_2S_2O_3$ were added at concentrations of 0.1 mM and 10 mM respectively. Cell density was estimated from the OD at 562 nm. ERG in the cultured supernatant was quantified by Sulfur index analysis described in Methods.

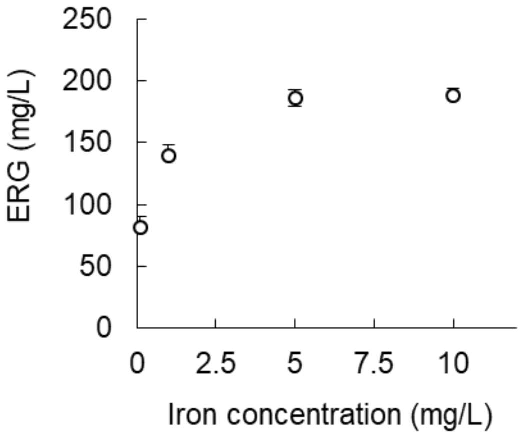

Figure S4. Effect of supplementation with iron on ERG productivity.

CH Δ*metJ* pQE1a-*egtABCDE* cells were cultured in SM1 liquid medium which contained AFC at various concentrations (0.1, 1, 5, 10 mg/L). After 6 h cultivation, IPTG and $Na_2S_2O_3$ were supplied at concentrations of 0.1 mM and 10 mM, respectively. ERG in the cultured supernatant at 120 h was quantified by Sulfur index analysis described in Methods.
